# Electric field assisted motion of a mercury droplet

**DOI:** 10.1038/s41598-020-80375-1

**Published:** 2021-02-02

**Authors:** Gábor Holló, Nobuhiko J. Suematsu, Elliott Ginder, István Lagzi

**Affiliations:** 1 MTA-BME Condensed Matter Physics Research Group, Budapest, Hungary; 2 School of Interdisciplinary Mathematical Sciences, Graduate School of Advanced Mathematical Sciences, and Meiji Institute for Advanced Study of Mathematical Sciences (MIMS), Meiji University, Nakano, Japan; 3 Department of Physics, Budapest University of Technology and Economics, Budapest, Hungary

**Keywords:** Chemistry, Physics

## Abstract

Field-assisted self-assembly, motion, and manipulation of droplets have gained much attention in the past decades. We exhibit an electric field manipulation of the motion of a liquid metal (mercury) droplet submerged in a conductive liquid medium (a solution of sulfuric acid). A mercury droplet moves toward the cathode and its path selection is always given by the steepest descent of the local electric field potential. Utilizing this unique behavior, we present several examples of droplet motions, including maze solving, electro-levitation, and motion on a diverted path between parallel electrodes by controlling the conductivity of the medium. We also present an experimental demonstration of Fermat's principle in a non-optical system, namely a mercury droplet moving along a refracted path between electrodes in a domain having two different conductivities.

## Introduction

Investigation into the self-propulsion of particles and droplets having various chemical and physical properties, as well as chemical compositions, has attracted much attention in the past several decades^1–8^. The gathered knowledge is fundamentally important and contributes to advancing the design of self-propelled systems with wide capabilities. These systems can be used in a diverse range of applications, including in medicine (e.g., targeted drug delivery)^9–15^ and environmental remediation^16,17^. The motion of these entities is generated and limited at either the liquid/air, liquid/solid interfaces^18^, or in the bulk liquid phase^19–21^. In most cases, the common characteristic is that the driving force of the self-propelled motion originates from interfacial tension differences which create and maintain Marangoni flows in the environment, or within the droplets^22,23^.

Response to external stimuli is a useful function of self-propelled objects, and a variety of such properties have been realized. Chemotaxis^24–27^, phototaxis^28,29^, magnetotaxis^30–33^, and electrotaxis^34^ are representative phenomena, and environmentally responsive artificial objects have even demonstrated their ability to reach target areas^25^, and to solve certain mathematical problems^35^.

Liquid metal droplets, such as those composed of mercury (Hg) or gallium, within a conductive liquid environment (a solution of sulfuric acid, sodium hydroxide) and in the presence of a direct electric field exhibit self-propelled motion towards an electrode^36–38^. The main aim of this study is to systematically investigate and understand the electric field assisted self-propelled motion of a Hg droplet in a sulfuric acid solution, and to illustrate several uses of this unique property. Our applications include maze solving in 2 and 3 dimensions, electro-levitation, path selection of a Hg droplet directed by the gradient of the electric field potential, and droplet manipulation in a non-homogeneous conductivity media. Surface tension driven phenomena related to Hg droplets (e.g., mercury beating heart)^39,40^ and their corresponding (electro)chemistry are well understood^41^, and we recall that Hg droplets have been used in polarography as working electrodes (the dropping mercury electrode)^42^.

## Results and discussion

### Motion in a capillary tube

**Figure 1:**
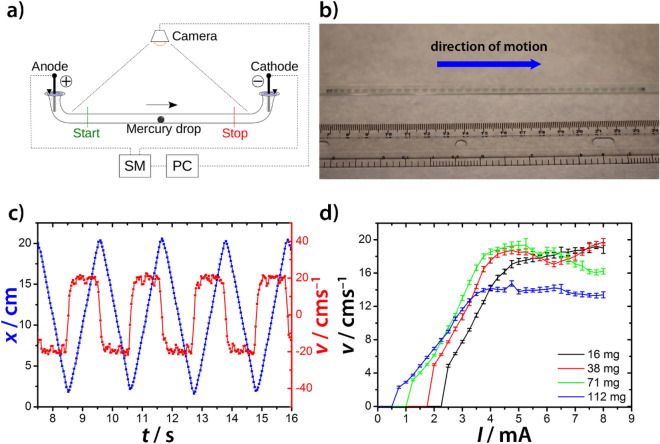
(**a**) The sketch of the experimental setup to investigate the motion of a Hg droplet in a direct electric field. (**b**) The motion of a Hg droplet (*m *= 38 mg) in a direct electric field using the galvanostatic condition (*I* = 3.0 mA) and a capillary tube (diameter of 3.3 mm) filled with the solution of sulfuric acid (*c* = 25.0 mM). The superimposed image shows the position of the droplet every 0.04 s. (**c**) The position-time graph of the Hg droplet (blue) and the velocity of the droplet (red). The droplet exhibits uniform motion. The direction of the electric field was changed whenever the droplet arrived near the cathode. (**d**) Velocity dependence of the Hg droplet for various sizes (masses) on the applied direct electric current in the solution of the sulfuric acid (*c* = 25.0 mM).

Capillary tubes and channel networks with different complexities were filled with a solution of sulfuric acid, and a droplet of liquid mercury was placed in the solution. An electric field was generated and maintained by a current generator using either copper or graphite electrodes. In all experiments, we used galvanostatic conditions instead of potentiostatic ones, because they provides a better estimate of the potential drop on the Hg droplet. In the presence of an electric field, the Hg droplet started to move toward the cathode with a uniform motion (Fig. 1a–c)^37^. This motion can be reversed by periodically changing the direction of the electric field (see Supplementary Movie S1). The mechanism of the droplet’s self-propelled motion is the following. In the presence of an electric field, the Hg droplet in the solution of sulfuric acid is negatively charged^39^, and a interfacial tension difference is generated at the interface between the liquid mercury and the sulfuric acid solution based on the Lippmann equation:

1 $$\frac{\partial \gamma }{{\partial \varphi }} = - \vartheta ,$$

where $\gamma$, $\varphi$, and $\vartheta$ denote the interfacial tension of the mercury, electric field potential and the surface charge density on the droplet, respectively^43^. This phenomenon is known as the electrocapillary effect^36,37^. Based on the Lippmann equation, the interfacial tension generated in an electric field at the liquid (mercury)/liquid (sulfuric acid solution) interface on the side of the droplet facing the anode is higher than the interfacial tension on the side of the droplet facing the cathode. This interfacial tension difference induces a Marangoni flow on the surface of the Hg droplet from the cathodic side (lower interfacial tension region) to the anodic side (higher interfacial tension region) of the droplet, which is simultaneously compensated by a bulk flow inside the droplet in the opposite direction. The overall effect is a displacement of the droplet toward the cathode.

Another important aspect of the motion is that there is a threshold electric potential difference dropping on the Hg droplet (${\Delta} \varphi$) above which the droplet starts to move. The manifestation of this behavior is that there is a threshold electric current for the motion observed in experiments, since the voltage applied between the electrodes (*U*) is proportional to the electric current (*I*) and ${\Delta} \varphi \sim {U}$, thus ${\Delta} \varphi \sim{I}$. The threshold current depends on the size of the droplet (Fig. 1d) and the concentration of the sulfuric acid (see Supplementary Fig. S1). In particular, we find that increasing the size of the droplet lowers its corresponding threshold current. Supplementary Movie S2 shows this counterintuitive behavior, in which the droplet attached to the bottom of a glass surface. At high electric currents both Hg droplets start to move, however, at lower currents, the larger droplet moves toward the cathode, while the smaller one remains at rest. This phenomenon, and the existence of the threshold potential difference, can be explained by contact angle hysteresis^44^. Differences in contact angles are caused by the electrocapillary effect, which changes the interfacial tensions at the sides of the Hg droplet facing the electrodes. The droplet remains at rest until the force generated by the interfacial tension difference overcomes the adhesive force corresponding with the contact angle hysteresis. The three-phase-contact line would not move if the value of the contact angle is between $\theta_{R}$ and ${\theta }_{A}$, where $\theta_{R}$, and ${\theta }_{A}$ are receding and advancing contact angles, respectively. Here, we consider a case, in which the threshold value of ${\Delta} \varphi$ drops on the Hg droplet. Therefore, the contact angles ${\theta }_{+}$ and ${\theta }_{-}$ are equal to ${\theta }_{R}$ and ${\theta }_{A}$, respectively, where ${\theta }_{+}$ and ${\theta }_{-}$ are contact angles in anodic and cathodic sides of the droplet, respectively. Assuming that the interfacial tensions between solid (surface) and Hg and solid (surface) and solution are constant, the following equation can be obtained by Young equations at the edges facing anode and cathode

2 $$\gamma_{ + } \cos \theta_{R} = \gamma_{ - } \cos \theta_{A} ,$$

where ${\gamma }_{+}$ and ${\gamma }_{-}$ are interfacial (Hg and solution) tension facing to anode and cathode and ${\gamma }_{+}>{\gamma }_{-}$ due to electrocapillary effect. Advancing and receding contact angles, ${\theta }_{R}$ and ${\theta }_{A}$ are negatively and positively deviated value from the equilibrium contact angle, ${\theta}_{E}$. If the value of the deviation, ${\Delta} \theta$, is much smaller than ${\theta }_{E}$, cosine in Eq. (2) can be approximated as follows

$$\left\{ {\begin{array}{*{20}c} {\cos \theta_{R} = \cos \left( {\theta_{E} - {\Delta} \theta } \right) \cong \cos \theta_{E} + {\Delta} \theta \sin \theta_{E} } \\ {\cos \theta_{A} = \cos \left( {\theta_{E} + {\Delta} \theta } \right) \cong \cos \theta_{E} - {\Delta} \theta \sin \theta_{E} } \\ \end{array} } \right.$$

Based on the linearization, Eq. (2) can be rewritten to the following equation.

$$\left( {\gamma_{ + } + \gamma_{ - } } \right){\Delta} \theta \sin \theta_{E} = - \left( {\gamma_{ + } - \gamma_{ - } } \right)\cos \theta_{E} ,$$

By assuming that the interfacial tension difference under the condition of bifurcation point of droplet motion (${\Delta} {\gamma }^{*}={\gamma }_{+}-{\gamma }_{-}$), is much smaller that the natural value of interfacial tension, ${\gamma }_{0}$, $\left({\gamma }_{+}+{\gamma }_{-}\right)$ can be approximated by $2{\gamma }_{0}$. Therefore, the threshold of the interfacial tension difference, ${\Delta} {\gamma }^{*}$, can be expressed as follows

$${\Delta} \gamma^{*} = - \frac{{2\gamma_{0} \sin \theta_{E} }}{{\cos \theta_{E} }} {\Delta} \theta ,$$

where ${\cos \theta_{E} }$ is negative constant due to ${\theta }_{\mathrm{E}}>\frac{\uppi }{2}$, and thus, ${\Delta} {\gamma }^{*}$ and ${\Delta} \theta$ are positive values. In the equation, the right-hand side is constant. Thus, the threshold value of the interfacial tension difference for propelling the droplet is independent of the size and current. From the Lippmann equation (Eq. 1), we get ${\Delta} \gamma \sim {\Delta} \varphi$ and the potential difference dropping on the droplet is proportional to its size and the electric current (${I}$), ${\Delta} \varphi \sim {rI}$, thus ${\Delta} \gamma \sim {rI}$. Therefore, the necessary condition for the droplet motion is ${rI}>{\Delta} {\gamma }^{*}$. This is an explanation of why a smaller droplet requires high current to start moving. Supplementary Fig. S2 presents the shape of the droplet, the corresponding contact angles and forces generated by the electrocapillary effect and contact angle hysteresis.

At values greater than the threshold current, we remark that the droplet experiences uniform motion. Its velocity is linearly proportional to the applied current, and at higher electric current the velocity saturates (Fig. 1d and Supplementary Fig. S1). The linear dependence can be theoretically explained by the following argument. The force generated due to the interfacial tension difference (${F}_{\gamma }$) is proportional to ${\Delta} \gamma$. Based on the Lippmann equation (Eq. 1), we get ${F}_{\gamma } \sim {\Delta} \varphi$ and ${\Delta} \varphi \sim {rI}$, thus ${F}_{\gamma }\sim {rI}$. By Stoke’s law the drag force ( ${F}_{\mathrm{d}}$ ) is proportional to ${rv}$, where ${v}$ is the velocity of the droplet. In the case of uniform motion, ${F}_{\gamma }={F}_{\mathrm{d}}$, the velocity of the droplet depends linearly on the electric current (${v}\sim I,$ Fig. 1d and see Supplementary Fig. S1). It is also evident that the velocity depends linearly on the electric potential difference dropping on the Hg droplet, (${v}\sim {\Delta} \varphi$). Therefore, the motion of the Hg droplet can be better described by the (local) electric current (determined by the gradient of the electric potential) rather than by the voltage applied between the electrodes. Of course, in a medium with a homogeneous electric field, the current (and the electric potential drop on the droplet) is proportional to the voltage, and thus the applied voltage can be just as useful as the current when quantifying the motion.

Knowing the conductivity of the sulfuric acid solution (Supplementary Fig. S3)^45^, the applied electric current, and the size of the droplet enabled us to estimate the electric potential difference drop across the Hg droplet (Supplementary Fig. S4). Supplementary Figure S5 shows the velocity dependence of the Hg droplet on the estimated electric potential difference drop of the droplet for various sizes (mass) and concentrations of the sulfuric acid. Similar to the prediction that ${v}\sim {\Delta} \varphi$, and we observed that the estimated threshold potential difference only slightly depends on the size of the droplet and on the concentration of the sulfuric acid. This behavior is vividly illustrated through changing the concentration of the sulfuric acid (see Supplementary Fig. S1 and S5b). To provide a better estimation of this threshold potential drop from the velocity and electric current curves (Fig. 1d and Supplementary Fig. S1), we estimated the threshold electric current for initiating of the motion (*I*_v=0_) as the function of the sulfuric acid concentration and size of the droplet (Supplementary Fig. S6). The threshold electric current was estimated as an intercept of the linear function fitted on the linear regimes of the velocity-current curves with the axis *I*. Supplementary Figs. S1 and S6a present that to initiate Hg droplet motion in a concentrated sulfuric acid solution a greater current is needed. This observation can be explained by the following arguments. The potential drop on the Hg droplet depends on the potential drop in the electrolyte solution, which is proportional to the applied voltage. To reach the threshold potential drop on the Hg droplet to initiate its motion, the same voltage should be applied between the electrodes irrespective of the conductivity (concentration) of the sulfuric acid solution. However, it should be noted that in very diluted solutions the voltage applied might be increased to initiate the droplet motion. In this case, the exchange current density is small at the electrodes, which generates greater potential drop on the electrodes (compared to the cases in which more concentrated electrolyte solutions are used) thus generating a lower potential drop on the electrolyte medium and Hg droplet. The same voltage generates a greater electric current in a more conductive (less resistive) medium than in a less (more resistive) conductive one (since *I* = *U*/*R*, where *R* is the electric resistance), and the electric conductance (the electric resistance) of the sulfuric acid solution increases (decreases) with its concentration. An opposite trend can be observed in the dependence of *I*_v=0_ on the droplet size (Fig. 1d and Supplementary Fig. S6b), a bigger droplet needs a smaller eclectic current, which can be explained by the fact that the potential drop across the droplet scales with its diameter. From the conductance of the sulfuric acid solution and *I*_v=0_ data, the obtained estimated value of the threshold potential drop across the Hg droplet is 0.28 ± 0.08 V, which is, as we discussed earlier, independent of the sulfuric acid concentration and the droplet size.

### Motion in a channel network with a uniform channel width: maze solving

**Figure 2:**
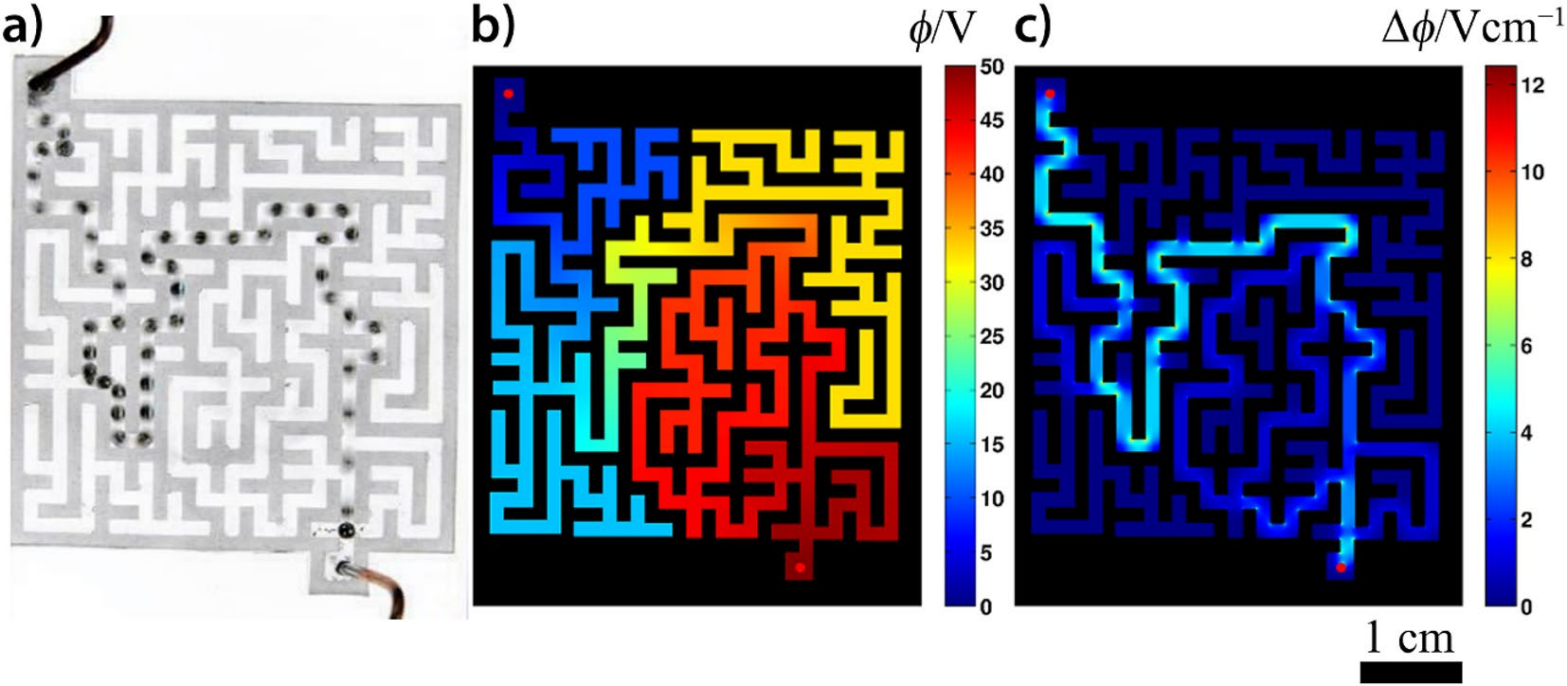
(**a**) Solution of a two-dimensional maze made of silicone using a Hg droplet (*m* = 20 mg) in an electric field using the galvanostatic condition (*I* = 5.0 mA). Two electrodes were placed at the entrance (anode) and exit (cathode) of the maze filled with the solution of sulfuric acid (*c* = 10 mM). The depth of the solution in the maze was ~ 1 mm. The superimposed image shows the position of the droplet every 0.04 s. (**b**) The calculated electric field and (**c**) the magnitude of the gradient in the maze. The steepest descent of the electric field is along the shortest path within the maze, which was followed by the droplet.

As an application of this behavior, we show that an electric field can be used to guide a Hg droplet through a maze filled with a sulfuric acid solution. When a high enough voltage was applied between electrodes placed at an entrance (anode) and an exit (cathode), a Hg droplet placed near to the entrance moved through a complex maze with a velocity of ~ 20 cm s^−1^ (Fig. 2a and see Supplementary Movie S3). This technique is one of the most powerful approaches for solving complex mazes because the velocity of the droplet does not depend on the complexity of the channel network. We also remark that the time required to obtain the solution is comparable with the time required when using glow discharge in a microfluidic chip filled with helium^46^. Other techniques can provide the solution in times ranging from several seconds (pressure-driven maze solving)^47^, to several hours (fungi assisted maze solving)^48^. In our maze, the Hg droplet travels along the path of shortest distance, and it is natural to assume that this path corresponds to the gradient flow of the electric potential. To illustrate this, we calculated the electric potential and its gradient for this particular maze (Fig. 2b,c). It can be seen that the path of steepest descent, in the maze topology, is indeed along the shortest path. In particular, the direction of its motion is driven by the gradient of the electric field potential (the droplet moves against the gradient). It should be noted that our maze is topologically very simple (even if it looks complicated at first glance). This is due to the fact that at each junction there are only two branches with a shorter and longer path (or paths with a dead end). Although the Hg droplet moves along the shortest path in this maze, we will shortly see that, in topologically more complex channel networks, the droplet can move along paths with greater distances, and that the path selection is driven by the local gradient of the electric field.

**Figure 3:**
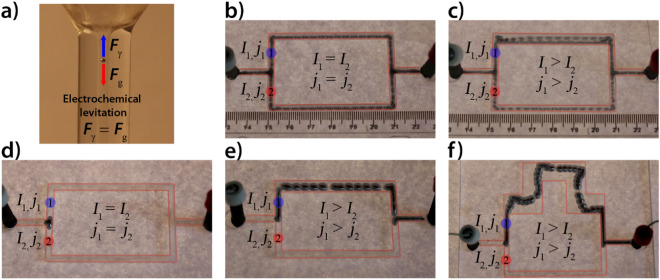
(**a**) Electro-levitation of a Hg droplet (*m* = 6 mg) in a vertically oriented capillary tube filled with the solution of sulfuric acid (*c* = 6.25 mM). The inner diameter of the capillary tube is 1.9 mm. Stochastic motion of a smaller Hg droplet (*m* = 20 mg) in an electric field using the galvanostatic condition (*I* = 3.0 mA) in channel networks filled with a solution of sulfuric acid (*c* = 25 mM) having the same lengths (*L*_1_ = *L*_2_ = 12 cm): (**b**) same widths (*w*_1_ = *w*_2_ = 3 mm) and (**c**) different widths (*w*_1_ = 6 mm, *w*_2_ = 3 mm). Deterministic motion of a larger Hg droplet (*m* = 350 mg) in an electric field using the galvanostatic condition (*I* = 3.0 mA) in channel networks having the same lengths (*L*_1_ = *L*_2_ = 12 cm): (**d**) same widths (*w*_1_ = *w*_2_ = 3 mm) and (**e**) different widths (*w*_1_ = 6 mm, *w*_2_ = 3 mm). (**f**) The motion of the same Hg droplet in a channel network filled with a solution of sulfuric acid (*c* = 25 mM) having different lengths and widths (*L*_1_ = 16 cm, *L*_2_ = 12 mm, *w*_1_ = 12 mm, *w*_2_ = 3 mm). The depth of the solution in the channel network was ~ 4 mm. Superimposed images show the position of the droplet every 0.04 s.

Since the motion of the Hg droplet in an electric field is not limited to liquid/solid or liquid/gas interfaces, the droplet can be levitated in a vertically oriented capillarity tube for specific electric currents, i.e., when the interfacial tension force originating from the electric field acting on the droplet is in balance with the gravitational force (Fig. 3a and Supplementary Movie S4). These observations lead us to use this setup to solve 3D mazes. Supplementary Movie S4 also illustrates how an electric field can be used to guide the Hg droplet within a vertically and horizontally connected capillarity network.

### Motion in a channel network with a non-uniform channel width

To get more insight into the complex motion of our Hg droplet, we carried out experiments in topologically simple channel networks consisting of two branches with common T-junctions near the cathode and the anode. We had three types of networks. In the first, the lengths and the widths of the branches were identical. In the second, the lengths were the same, but their widths were different. In the third, the wider branch was prescribed a longer length. It should be noted that at T-junctions the widths of the channels were the same. We used two different Hg droplets, a smaller droplet (*m* = 20 mg) with a size smaller than the width of the channel, and a bigger droplet (*m* = 350 mg) which tightly fit within the channel filled with the sulfuric acid solution.

When we used the first template (identical channel lengths and widths), the path selection of the smaller Hg droplet in the T-junction was approximately 50%:50% based on the analyzed motion (Fig. 3b and Supplementary Movie S5). In this case, both the electric current and current density were the same, and therefore the direction of the motion at the T-junction was determined by a stochastic effect attributed to the impact of the droplet on the wall of the channel. When we used the second template (identical channel lengths with different widths), the droplet favored moving into the branch with a wider width (less resistance) at a path selection rate of 90%:10% (Fig. 3c and Supplementary Movie S5). This finding can be explained by the fact that the electric currents at the two branches are different. That is, since they have different resistances and the widths of the channel at the junction were the same, the droplet experienced higher and lower current densities and preferentially moves in the direction of the higher current density. Nevertheless, the Hg droplet sometimes moved towards the lower current density branch due to the stochasticity imparted by the impact of the droplet with the wall. This behavior highlights a very important aspect of the motion, namely, that once the motion of the droplet is determined, it continues its motion toward the cathode and that the motion is determined by the local electric field. This describes our finding that once the droplet moved into the region of lower current density, it does not return and continues to follow the electric field lines.

The behavior of the motion of a small Hg droplet can be described as a stochastic motion because the path selection is determined by two factors: the difference in the local current density (determined by the structure of an electric field) and the effect of the impact of the droplet with the wall at the T-junction. To eliminate the later effect, we used a larger Hg droplet that fully accommodated the T-junction when it started to move toward the cathode. This setup enables us to understand more deeply the path selection of the droplet. When the first type of network was used, the droplet arrived at the T-junction, but after this point the droplet remained at rest and no further motion was observed (Fig. 3d and Supplementary Movie S6). Only a small shape deformation was detected at this stage. In the second network, the droplet stayed for a while at the junction before proceeding to move into the branch having the greater current density—less net resistance (Fig. 3e and Supplementary Movie S6). This behavior persisted, and the droplet never returned into the branch with lower current density (higher resistance). In other words, compared with the motion of the smaller droplet in the same channel network, the motion, in this case, was deterministic.

In order to understand the behavior of the larger droplet, one should take into account that the widths of the branches at the T-junctions had the same size. In the first type of network, when the larger Hg droplet accommodated the junction, two of its sides point toward the cathode, while the middle part is closer to the anode. In this orientation, two interfacial tension driven forces acted on the droplet. Their directions were opposite and the forces were balanced because the current density was identical in the two branches (locally the gradients of the electric field were the same). Therefore, the droplet did not move. When the current densities (gradients of the electric potential) were different, the resultant force pointed towards the region of higher density (higher local gradient of the electric potential) and the droplet started to move in that direction.

More interestingly, we also investigated the motion in the third type of network (one branch having a wider and longer channel). In this case, the droplet moved along the longer path (Fig. 3f and Supplementary Movie S6), i.e., the droplet did not choose the path with the shortest distance. It moved in the direction of lower resistance (higher local current density, higher absolute value of the local gradient of the potential) in the T-junction, and this observation highlights a very important issue, namely, that the path selection of the Hg droplet in an electric field is driven by the local gradient of the electric field, and that the droplet moves toward the steeper local gradient of the electric potential. This is because the motion is driven by the net force acting on the Hg droplet, and a greater force is generated on the side of the droplet facing a steeper gradient of the electric field potential (higher current density, lower net resistance). That is, a steeper local gradient of the potential generates a greater potential drop and a correspondingly larger interfacial tension driven force acting on the droplet. We note that, the direction of the local gradient does not always point along the shortest path.

**Figure 4:**
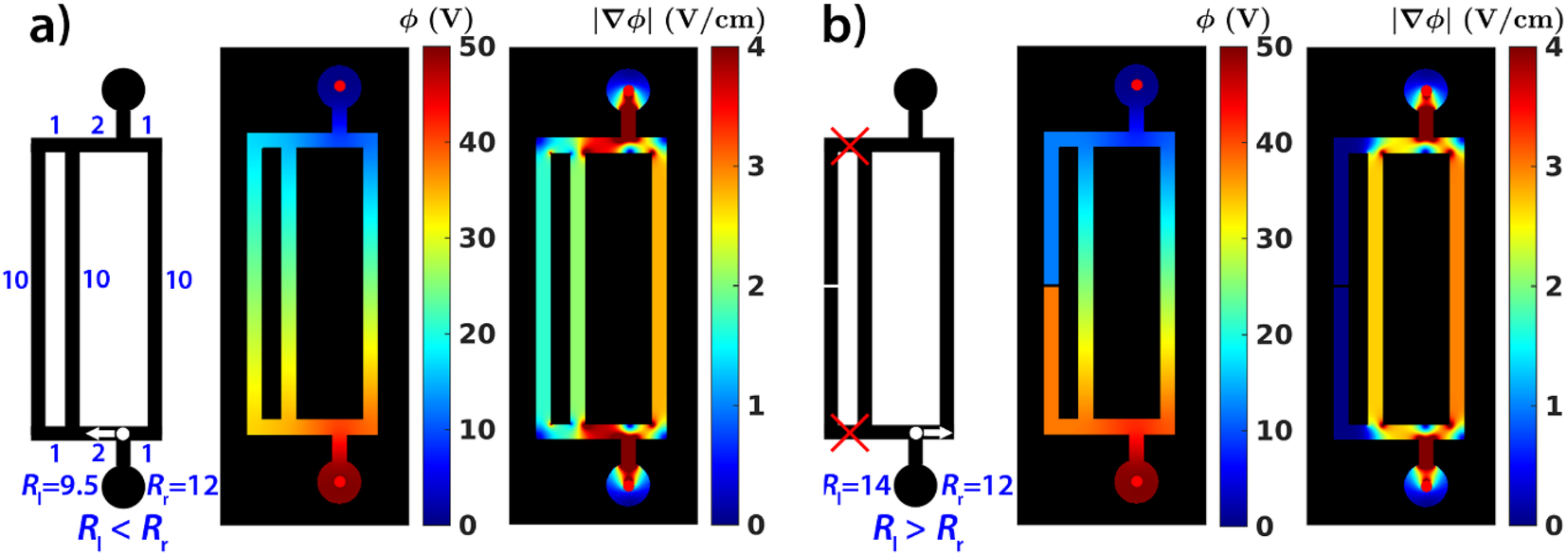
Path selection of a Hg droplet in two channel networks in the presence of an electric field, showing the network topology, calculated electric field potential and the magnitude of the gradient. (**a**) The T-junction near the anode has two outlets: one having a longer and parallelly connected paths (left side) and another single shorter path (right side). The net resistance of the left side of the network is smaller than that on the right. (**b**) The T-junction near the anode has two outlets: one with a single longer path (left side) and another with a shorter (right side) path. The net resistance of the left side of the network is greater than that on the right. The droplet moves in the locally lower resistance branch irrespective of the path lengths.

To highlight this very important principle of path selection in channel networks, we simulated the electric field in a relatively simple setup. The configuration consisted of three paths with uniform widths offering three possible choices for the motion of the Hg droplet (Fig. 4). A T-junction near the anode has two outlets; one with a longer and parallelly connected region (left side, Fig. 4a) and another with a single shorter path (right side, Fig. 4a). In this setup, at the T-junction near the anode, our computations show that the droplet would move towards the parallelly connected paths, thus moving not along the shortest path. Again, this is because the gradient of the potential in the T-junction is steeper (the current density is higher) towards the parallelly connected paths (Fig. 4a). This can be rephrased to say that the droplet prefers to move in the direction of the branch with a locally lower resistance. From the structure of this particular channel network it is evident (knowing that the resistance is proportional to the length of the channel) that the net resistance on the left side of the network (even if they have longer lengths) is smaller than that on the right. This is because, in a parallel circuit, the net resistance decreases as more components are added (Fig. 4a). However, when one component of the parallelly connected channels is removed resulting in two paths, the gradient of the potential in the T-junction is steeper (the current density is higher) towards the shortest path (Fig. 4b). Therefore, in this setting, the Hg droplet would move along the path with the shortest distance (Fig. 4b). In such a network it is trivial that the resistance of the right side (shorter length) of the network is lower than that of the left (longer length). This simple argument points out the fact that, in a complex channel network, droplets would not always move along the shortest distance, because its path selection is based on the local gradient of the electric potential.

### Motion in a non-homogeneous conductive medium

**Figure 5:**
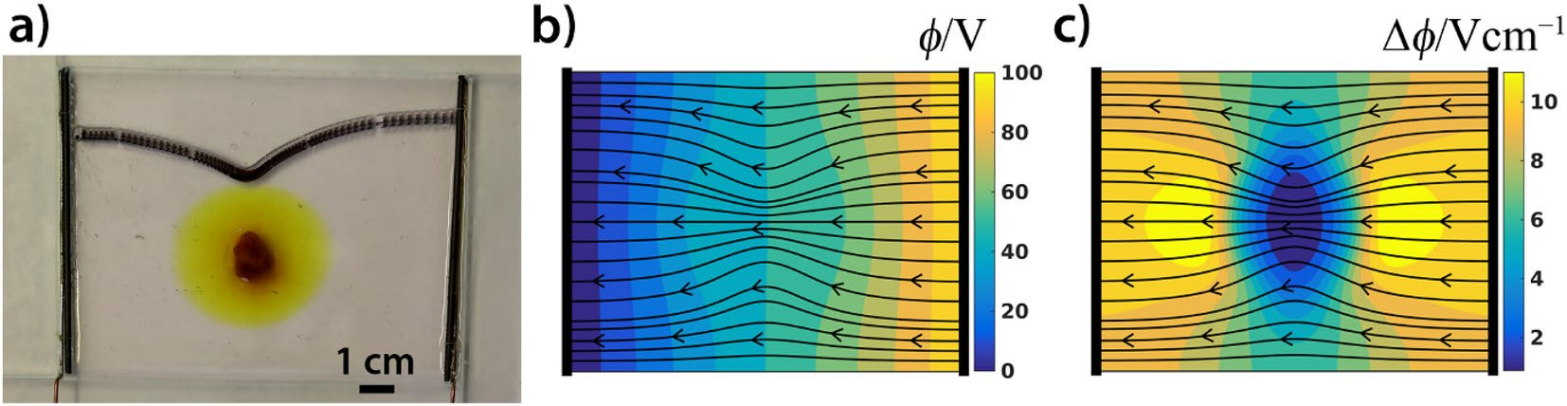
(**a**) Path deflection of a Hg droplet (*m* = 200 mg) moving in an electric field using the galvanostatic condition (*I* = 3.0 mA) between the parallel electrodes in a solution of sulfuric acid (*c* = 25 mM). The domain was filled with distilled water and a piece of iron (III) chloride (FeCl_3_) crystal was placed in the center of the container. The experiment started when the dissolved salt created a radially symmetric gradient of ions around the crystal. The depth of the solution in the domain was ~ 5 mm. The superimposed image shows the position of the droplet every 0.04 s. (**b**) The calculated electric field and (**c**) the magnitude of the gradient with the corresponding electric field lines.

To illustrate the impressive power of Hg droplet manipulation by an electric field, we also provide an example where the Hg droplet was forced to move on a diverted path between parallel electrodes in a non-homogeneous conductive medium. In a homogeneous conductive media, it is clear that the path of the droplet will be along a straight line. We created a non-homogeneous conductive medium by placing crystals of iron chloride (FeCl_3_) in distilled water. The water-soluble iron chloride dissociated into ions of Fe^3+^ and Cl^−^, which radially diffused to create a concentration gradient of ions (conductive species). This established a spatially non-homogeneous conductive domain around the solid crystals. When an electric field was applied between the electrodes, the Hg droplet moved on a deflected path. We observed that its motion curves towards the solid crystals while continuing to move toward the cathode (Fig. 5a and Supplementary Movie S7). We remark that this finding resembles the nature of gravity, namely the gravitational force is a manifestation of the curved space–time induced by masses. This effect causes the deflection of the light near large masses due to the distortion of the time–space. In our case, the fact that the Hg droplet moves towards the solid crystal is a manifestation of the fact that the dissolved ions change the local conductivity of the medium, thus changing the structure of the electric field. Here the structure of the electric field (electric field strength) is determined by the concentration of the ions (conductivity) and the shape and position of the electrodes. Numerical simulations of the electric field potential and its gradient support our experimental observations (Fig. 5b,c). The direction of the droplet’s motion is along the gradient of the electric potential (the direction of the electric current density). In other words, the Hg droplet moves along the electric field lines, where the direction of its motion points towards the direction of the steepest descent of the electric potential, since $\overrightarrow{E}=-\nabla \varphi$.

### Fermat's principle in a non-optics system

**Figure 6:**
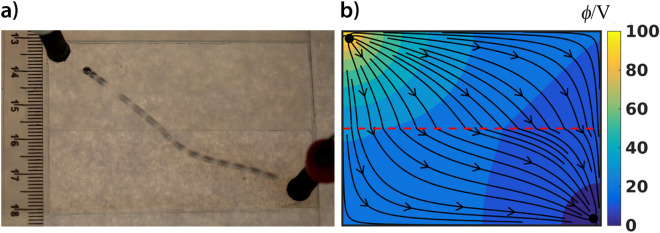
Refraction of the path of a Hg droplet (*m* = 80 mg) moving in an electric field through a boundary separating two different conductivity regions using the galvanostatic condition (*I* = 25 mA). The domain was filled with a solution of sulfuric acid (*c* = 25 mM). The depths of the solution were 2 mm (upper part) and 3 mm (bottom part). The superimposed image shows the position of the droplet every 0.04 s. The droplet in the lower conductive (higher resistance and current density) region moved faster than in the higher conductive (lower resistance and current density) region (**b**) The calculated electric field in the domain having two different conductive parts with the corresponding electric field lines.

The well-known Fermat's principle in optics says that the path taken by a ray between two given points is the path that can be traversed in the least time. The manifestation of this principle is the reflection and refraction of rays. Refraction occurs when a light ray travels between two points in media with different indices of refraction (i.e., different velocities). Experimental demonstration of Fermat’s principle in non-optical systems is lacking and challenging. In an animate system, for example, the members of an ant colony can be shown to follow Fermat's principle by forcing them to move on two different surfaces: one where they walked faster than another^49^. The path of a Hg droplet in the presence of an electric field is also refracted, when it travels through a boundary separating two domains with different current densities/conductivity (Fig. 6 and Supplementary Movie S8). To create such a medium, we designed a plexiglass container with different depths. Therefore, the conductivity/resistance of the sulfuric acid solution changes instantly at the boundary separating the two depths of the solution (see Supplementary Fig. S7a). Figure 6 shows the observed refraction of the Hg droplet and the calculated electric field with its corresponding field lines. Due to the inertia of the Hg droplet, there is no instantaneous change in the direction of the motion of the droplet at the boundary. Similar to the previous case, it can be seen that the droplet follows the electric field lines, which corresponds to the path of steepest descent of the electric field potential.

There is an obvious difference between our droplet system and the optic system, namely, in optics the angle of incidence is greater than the angle of refraction whenever light travels from a medium having greater phase velocity to a medium having a smaller phase velocity. This behavior can be described quantitatively by Snell’s law. Here, however, we have the opposite behavior. That is, the angle of incidence is smaller than the angle of refraction whenever the droplet moves from a medium with a greater velocity to a medium with a smaller velocity. From the image sequence, we estimated the velocity of the droplet in the two domains with different conductivity/resistance, and we obtained different velocities (${v}_{1}=13.0$ cm s^−1^ (Fig. 6a—upper domain) and ${v}_{2}=11.8$ cm s^−1^ (Fig. 6a—bottom domain)).

As previously stated, Fermat’s principle does not provide the explanation of our system’s behavior, however, one can intuitively hypothesize a related minimalization problem. Namely, that the Hg droplet follows the path along which the net resistance is minimized. We can formulate the problem as the minimization of ${R}_{1}+{R}_{2}$ (see the Supporting Information and Fig. S7b). The result of this treatment is similar that of the Snell’s law:

3 $$\frac{1}{{h_{1} }}\sin \alpha = \frac{1}{{h_{2} }}\sin \beta \;{\text{or}}\;v_{1} \sin \alpha = v_{2} \sin \beta$$

where ${h}_{1}$, ${h}_{2}$ and ${v}_{1}$, ${v}_{2}$ denote the depths of the solution and the velocities of the Hg droplet, respectively.

## Conclusion

In this study, we have investigated the complex motion of a liquid metal droplet in a solution of sulfuric acid, in which the motion of the Hg droplet towards the cathode was generated and maintained by using a direct electric field. We have shown that the velocity of the liquid metal droplet is proportional to the magnitude of the potential’s gradient (electric current density) which determines the potential difference drop across the droplet. This drop in potential generates the Marangoni flow within the liquid, which is responsible for the tactic motion towards the cathode. The direction of the motion is always determined by the local steepest descent of the electric field potential (i.e., against the local gradient of the electric potential, the direction of the electric current density vector, or along the electric field lines). We have also shown that the direction of the motion can be manipulated by changing the conductivity of the medium.

We note that miniaturization at the micro- and nanoscales would be very challenging because micro droplets would need high electric current in the electrolyte solution, which would contribute to the appearance of side effects such as temperature increase due to the heat dissipation since *q* = *UIt*. On the other hand, the shape change of the Hg droplet controlled by an electric field could be utilized in designing fluidic mixer for electrolyte solutions in milli- or microfluidic devices. We anticipate that our results have implications in applications ranging from field-assisted droplet manipulation, to soft robotics^50,51^.

## Materials and methods

### Experimental

In our experiments, we used borosilicate capillary tubes with an inner diameter of 3.3 mm. The 2D maze was fabricated by photolithography and made of poly(dimethyl siloxane) (PDMS) with a channel width and depth of 1.4 mm and 1.0 mm, respectively. The channel networks were prepared from plexiglass with various channel widths, and the depth of the channel was 4 mm. Sulfuric acid (H_2_SO_4_) and mercury were purchase from Sigma-Aldrich and Reanal, respectively. Copper and graphite electrodes, and to maintain the direct electric field the Keithley 2410 current generator, were used. Experimental images and videos were captured by using a Canon EOS 70D digital camera.

### Mathematical modeling and numerical simulation

As described in the previous section, the Marangoni flow on the surface of the droplet gives rise to its motion. Our experimental findings show that the net force on the droplet results in its motion along the electric field. As the following description shows, this observation can be modeled as a gradient flow of the electric field potential.

Let $\varphi$ denote the electric field potential and consider the center of mass of the droplet, ${x}_{c}.$ The rate of change of this quantity in the direction $v\in {R}^{2}$ can be computed as follows:

$$\left. {\frac{{d\varphi \left( {x_{c} + \epsilon v} \right)}}{d\epsilon }} \right|_{\epsilon = 0} = \nabla \varphi \left( {x_{c} } \right) \cdot v$$

where denotes the standard inner product. Since $v$ is a direction vector, we may assume its magnitude is unity, $||v||=1$, and use the Cauchy-Schwartz inequality to estimate the magnitude of the maximum rate of change around ${x}_{c}$:

$${\left.\frac{d\varphi \left({x}_{c}+\epsilon v\right)}{d\epsilon }\right|}_{\epsilon =0}\le \Vert \nabla \varphi \left({x}_{c}\right)\Vert \cdot \Vert v\Vert =\Vert \nabla \varphi \left({x}_{c}\right)\Vert .$$

It follows that the direction of steepest increase is given by the gradient of the potential function at ${x}_{c}$. Therefore the motion of the droplet’s center of mass can be modeled by the following ordinary differential equation:

$$\left\{\begin{array}{ll}{\dot{x}}_{c}=-\nabla \varphi \left({x}_{c}\right)& \quad t>0\,\, {x}_{c}\in \Omega \\ {x}_{c}\left(t=0\right)={x}_{0} & \quad {x}_{0}\in \Omega \end{array}\right.$$

where ${x}_{0}$ specifies the location of the center of mass within the experimental domain $\Omega$ at the initial time. The properties of gradient systems are well understood, and it is clear that the motion of ${x}_{c}$ is along the electric field $\overrightarrow{E}=-\nabla \varphi$.

In order to simulate the electric field potential and its gradient, we numerically solved the following partial differential equation

4 $$\left\{ {\begin{array}{ll} {\nabla \sigma \cdot \nabla \varphi = 0} & \quad {\left( {x,y} \right) \in \Omega } \\ {\frac{\partial \varphi }{{\partial \nu }} = 0} & \quad {\left( {x,y} \right) \in \partial \Omega_{wall} } \\ {\varphi \left( {x,y} \right) = 0} & \quad {\left( {x,y} \right) \in \partial \Omega_{c} \left( {x,y} \right) \in \partial \Omega_{c} } \\ {\varphi \left( {x,y} \right) = \phi_{0} } & \quad {\left( {x,y} \right) \in \partial \Omega_{a} } \\ \end{array} } \right.$$

where $\Omega$ denotes the experimental domain, $\partial\Omega =\partial {\Omega }_{wall}\cup \partial {\Omega }_{c}\cup \partial {\Omega }_{a}$ its border, $\nabla$ is the gradient operator, $\partial \varphi /\partial \nu$ is the outer normal derivative, and $\sigma$ and $\varphi$ denote the conductivity and electric field potential, respectively. Here $\partial {\Omega }_{\mathrm{wall}}$ denotes the region of no-flux boundary conditions, whereas $\partial {\Omega }_{c}$ and $\partial {\Omega }_{a}$ designate regions of Dirichlet boundary conditions at the cathode and anode electrodes, respectively. We remark that Eq. (3) can be derived from three equations, $\nabla \cdot \overrightarrow{j}=0$ (continuation equation), $\overrightarrow{j}=\sigma \overrightarrow{E}$, and $\overrightarrow{E}=-\nabla \varphi$.

Equation (4) was solved in 2D using a transient method of lines. The equation was discretized on an equidistant rectangular grid with a grid spacing of $d=7.3\times {10}^{-6}$ m, and the resulting set of the ordinary differential equations (ODEs) was solved using the backward Euler method.

In the case of maze solving, the size of the grid was $7780d$×$6440d$, and the conductivity was held constant. To simulate the electric field in a non-homogeneous conductive medium, the size of the grid was $540d$×$720d$, with $d=1.67\times {10}^{-2}$ m and the conductivity depends on the concentration of the salt dissolved ($c$) in the water phase as follows:

$$\sigma = 1 + 10c.$$

In the above, the concentration of the dissolved salt was calculated separately by solving the diffusion equation:

5 $$\left\{ {\begin{array}{ll} {\partial_{t} c = D\nabla^{2} c} & \quad {t > 0,\left( {x,y} \right) \in \Omega } \\ {\frac{\partial c}{{\partial \nu }} = 0} & \quad {t > 0, \left( {x,y} \right) \in \partial \Omega } \\ {c\left( {t = 0,x,y} \right) = 0} & \quad {\left( {x,y} \right) \in \Omega } \\ \end{array} } \right.$$

where $D$ is the diffusion coefficient of dissolved salt. Equation 5 was solved also using a method of lines on a uniform rectangular grid, where the ODEs were integrated in time using the Crank–Nicolson method. The diffusion coefficient of the salt was set to 10^−9^ m^2^ s^−1^, which is a typical value used for small hydrated ions in a water phase. The calculated salt concentration at $t={10}^{5}$ s was used to express the spatially dependent conductivity of the medium.

To simulate the structure of the electric field in two domains having different conductivities, we used the following values, ${\sigma }_{1}=0.78$ and ${\sigma }_{2}=2.28$ on a grid with dimensions $540d$×$720d$ with $d=1.67\times {10}^{-2}$ m. The model equation in this setting is expressed:

6 $$\left\{\begin{array}{l}\nabla \sigma \cdot \nabla \varphi =0 \left(x,y\right)\in \Omega \\ \frac{\partial \varphi }{\partial \nu }=0 \left(x,y\right)\in \partial {\Omega }_{wall}\\ \left[\varphi \right]=0, \left[\sigma \frac{\partial \varphi }{\partial \nu }\right]=0 \left(x,y\right)\in \partial {\Omega }_{c}\cap \partial {\Omega }_{a}\\ \varphi \left(x,y\right)=0 \left(x,y\right)\in \partial {\Omega }_{c}\\ \varphi \left(x,y\right)={\phi }_{0} \left(x,y\right)\in \partial {\Omega }_{a}\end{array}\right.$$

where the experimental domain ${\Omega } = {\Omega }_{a} \cup {\Omega }_{c}$ is composed of the two regions with different conductivities:

$$\sigma \left( {x,y} \right) = \left\{ {\begin{array}{*{20}c} {\sigma_{a} \left( {x,y} \right) \in {\Omega }_{a} } \\ {\sigma_{c} \left( {x,y} \right) \in {\Omega }_{c} } \\ \end{array} } \right.$$

In Eq. (6), the notation $\left[ \cdot \right]$ expresses the jump in its quantity across the interface separating ${\Omega }_{1}$ and ${\Omega }_{2}$^52^. In particular, the physical implications of the interfacial boundary conditions in Eq. (6) are to designate continuity, and a flux balance across the interface.

## Supplementary Information

Supplementary Information.

Supplementary Movie 1.

Supplementary Movie 2.

Supplementary Movie 3.

Supplementary Movie 4.

Supplementary Movie 5.

Supplementary Movie 6.

Supplementary Movie 7.

Supplementary Movie 8.
