# Supplementary material for: Electric field assisted motion of a mercury droplet
Source: Sci Rep. 2021 Feb 2;11:2753. doi: 10.1038/s41598-020-80375-1 (PMC7854757; doi:10.1038/s41598-020-80375-1)
Supplement: Supplementary file 1 — Supplementary Information. [file 41598_2020_80375_MOESM1_ESM.pdf]

Supplementary Information

Electric field assisted motion of a mercury droplet

Gábor Holló[1], Nobuhiko J. Suematsu[2], Elliott Ginder[2], István Lagzi[1,3]

[1]MTA-BME Condensed Matter Physics Research Group, Budapest, Hungary

[2]School of Interdisciplinary Mathematical Sciences, Graduate School of Advanced Mathematical Sciences, and Meiji Institute for Advanced Study of Mathematical Sciences (MIMS), Meiji University, Nakano, Japan

[3]Department of Physics, Budapest University of Technology and Economics, Budapest, Hungary

Derivation of the modified "Snell's law"

We hypothesize that a Hg droplet follows the paths along which the net resistance has a minimum. We can formulate the problem as a minimization of the quantity $R_1 + R_2$, i.e., $\frac{d(R_1+R_2)}{dx} = 0$ (Supplementary Fig. S7). If we suppose that the droplet moves in linear paths in the two domains, and using the fact that the resistance is linearly proportional to the length (l) and is inversely proportional to the depth of the solution (h), i.e., ($R \sim l/h$), l_1 and l_2 can be expressed as $l_1 = \sqrt{a^2 + x^2}$ and $l_2 = \sqrt{b^2 + (d-x)^2}$. Distances, a, b, d, and x, can be seen in Supplementary Fig. S7. After derivation, we obtain $\frac{x}{h_1\sqrt{a^2+x^2}} + \frac{(d-x)}{h_2\sqrt{b^2+(d-x)^2}}$, and introducing the sine of the angles of incidence (α) and refraction (β), we obtain $\frac{1}{h_1}\sin\alpha = \frac{1}{h_2}\sin\beta$. Measuring the angles, α = 52° and β = 75°, and knowing that h_1/h_2 = 2/3 from the depths of the solution in the two domains (Supplementary Fig. S7a), we obtain values of 0.394 and 0.322 for the left and right-hand sides, respectively. The velocity of the Hg droplet is proportional to the current density, since the electric current does not change, it is constant irrespectively to the depths of the solutions. Therefore, the current density is inversely proportional to the depth of the solution, and we obtain $v \sim 1/h$. Based on this, we can rewrite $\frac{1}{h_1}\sin\alpha = \frac{1}{h_2}\sin\beta$ in the form of $v_1\sin\alpha = v_2\sin\beta$.

Supplementary figures

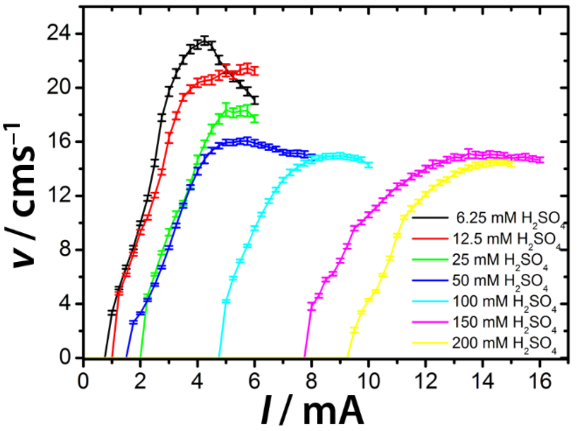

Supplementary Figure S1. Velocity dependence of the Hg droplet (m = 38 mg) on the applied direct electric current for various concentrations of the sulfuric acid solution.

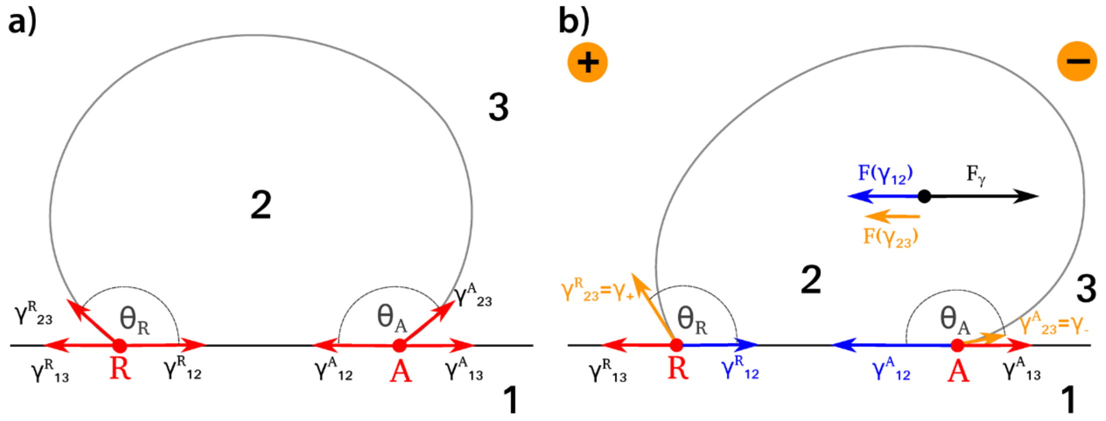

Supplementary Figure S2. Profiles of the droplet (a) in the absence and (b) presence of an electric field. γ_{13}, γ_{23} and γ_{12} are the solid surface free energy, liquid surface free energy, and solid/liquid interfacial free energy, respectively. θ is the contact angle; 1: solid, 2: liquid (mercury), 3: liquid (electrolyte solution, sulfuric acid solution). F_γ is the electrocapillarity force, and $F(\gamma_{12})$ and $F(\gamma_{23})$ are the contact angle hysteresis forces.

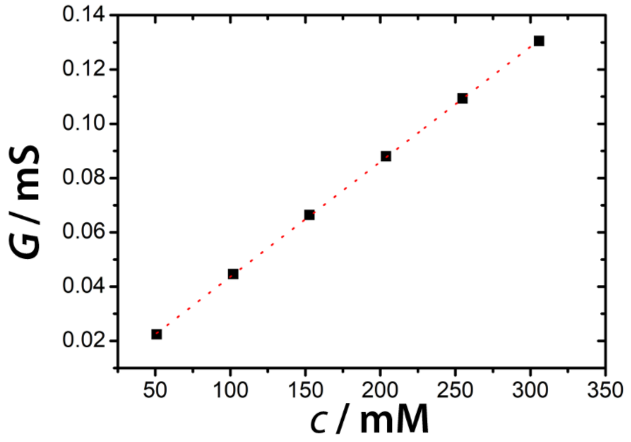

Supplementary Figure S3. Electric conductance of the sulfuric acid solution. Data were obtained from the work of Darling (Darling, H. E. Conductivity of Sulfuric Acid Solutions. J. Chem. Eng. Data 9, 421–426, 1964).

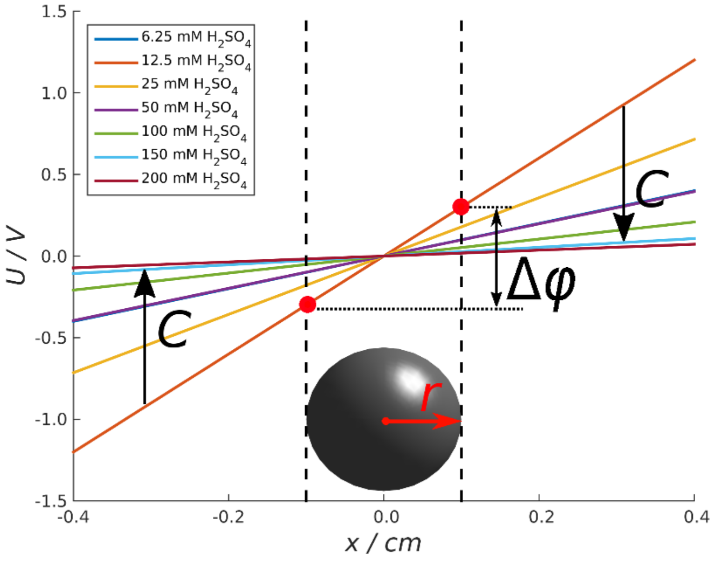

Supplementary Figure S4. The potential drop across the Hg droplet in an electric field at various concentrations of the sulfuric acid solutions. It can be seen that greater droplet size and smaller sulfuric acid concentration generate greater potential drop across the Hg droplet.

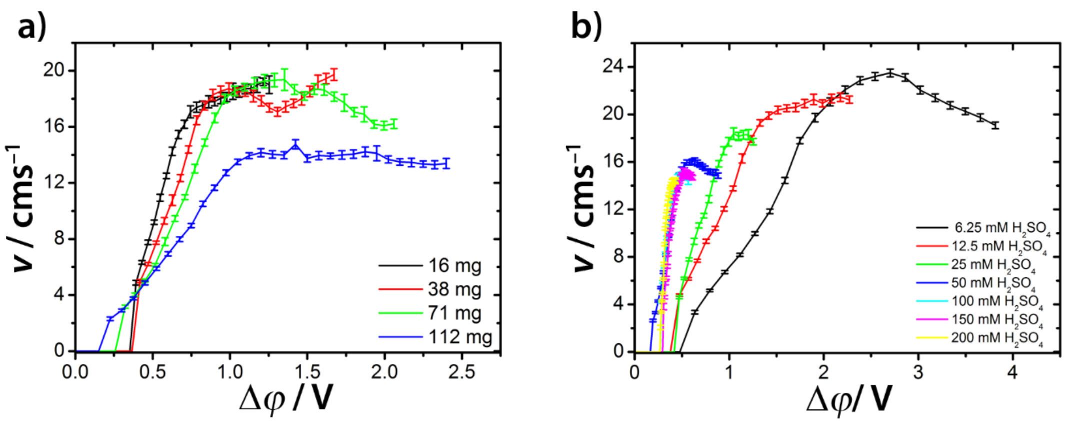

Supplementary Figure S5. Velocity dependence of the Hg droplet on (a) various sizes (masses) in the solution of the sulfuric acid (c = 25 mM) and (b) various concentrations of the sulfuric acid solution using a Hg droplet of m = 38 mg for the estimated potential drop on the two sides of the droplet in the electric field. The potential drop on the droplet ($\Delta\varphi$) is proportional to the applied electric field strength (E).

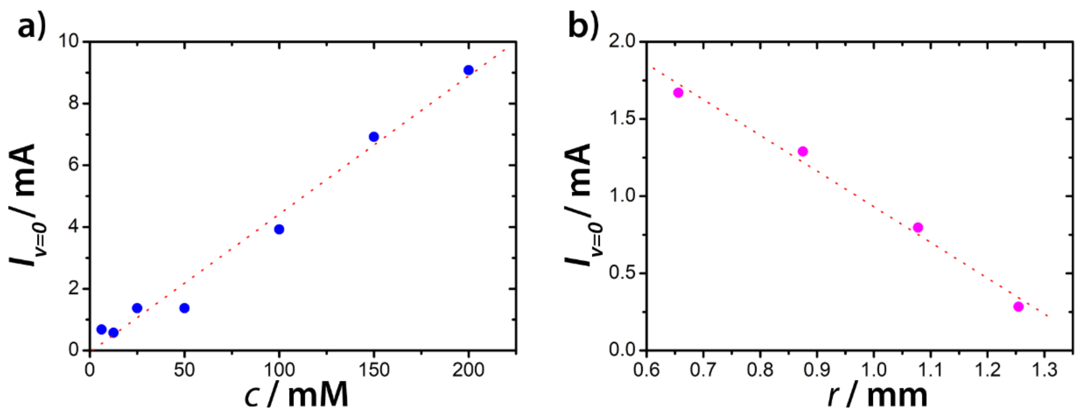

Supplementary Figure S6. Dependence of the threshold electric currents for the initiation of the motion of a Hg droplet on (a) the concentration of the sulfuric acid solution and (b) various sizes of the droplet. Data in (a) and (b) were determined from Supplementary Figure S1 and Fig. 1d, respectively.

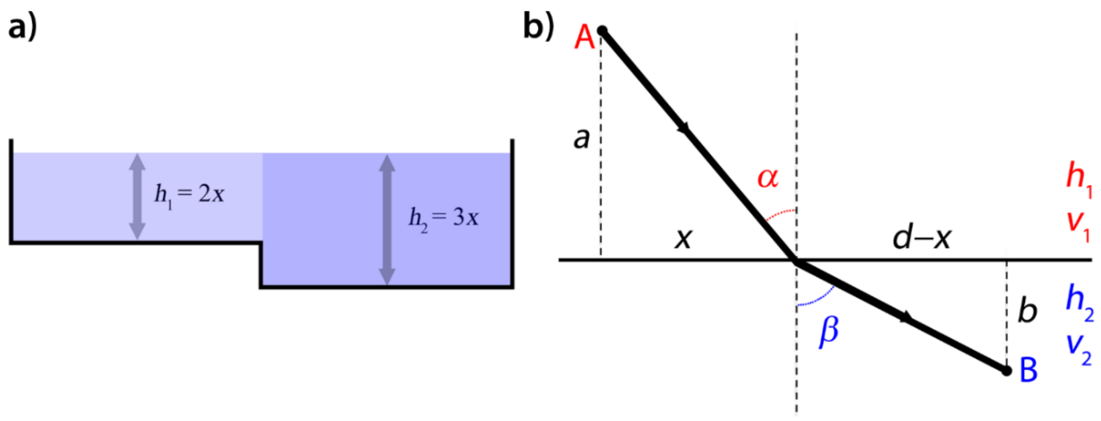

Supplementary Figure S7. (a) Cross section of the domain having different heights used in the refraction experiments of a Hg droplet filled with the solution of sulfuric acid. (b) The sketch (top view) showing the distances travelled by a Hg droplet in the solution of two different heights of sulfuric acid.

Supplementary movies

Movie S1. The motion of a Hg droplet (m = 38 mg) in a direct electric field using the galvanostatic condition (I = 3.0 mA) and a capillary tube filled with the solution of sulfuric acid (c = 25 mM). The droplet exhibits uniform motion. The direction of the electric field was changed whenever the droplet arrived near the cathode.

Movie S2. The effect of a direct electric field using the galvanostatic condition (I = 25 mA and I = 40 mA) on two Hg droplets (m_1 = 30 mg and m_2 = 790 mg) in the solution of sulfuric acid (c = 25 mM). The depth of the solution in the maze was ~ 4 mm.

Movie S3. Solution of a two-dimensional maze made of silicone using a Hg droplet (m = 30 mg) in an electric field using the galvanostatic condition (I = 5.0 mA). Two electrodes were placed at the entrance (anode) and exit (cathode) of the maze filled with the solution of sulfuric acid (c = 10 mM). The depth of the solution in the maze was ~ 1 mm. The droplet moves along the shortest path in the maze.

Movie S4. Electro-levitation and three-dimensional maze solving using a Hg droplet (m = 6 mg) using a direct electric field with the galvanostatic condition (I = 3.0 mA) in a vertically oriented capillary tube (diameter of 1.9 mm) filled with the solution of sulfuric acid (c = 6.25 mM).

Movie S5. Stochastic motion of a smaller Hg droplet (m = 20 mg) in a channel network filled with a solution of sulfuric acid (c = 25 mM) having the same length ($L_1 = L_2$ = 12 cm), same width ($w_1 = w_2$ = 3 mm) and the same length ($L_1 = L_2$ = 12 cm), different widths (w_1 = 6 mm, w_2 = 3 mm) in an electric field using the galvanostatic condition (I = 3.0 mA). The depth of the solution in the channel network was ~ 4 mm. The direction of the electric field was changed whenever the droplet arrived near the cathode.

Movie S6. Deterministic motion of a larger Hg droplet (m = 350 mg) in a channel network filled with a solution of sulfuric acid (c = 25 mM) having the same length ($L_1 = L_2$ = 12 cm), same width ($w_1 = w_2$ = 3 mm), the same length ($L_1 = L_2$ = 12 cm), different widths (w_1 = 6 mm, w_2 = 3 mm) and different lengths, widths (L_1 = 16 cm, L_2 = 12 cm, w_1 = 12 mm, w_2 = 3 mm) in an electric field using the galvanostatic condition (I = 3.0 mA). The depth of the solution in the channel network was ~ 4 mm. The direction of the electric field was changed whenever the droplet arrived near the cathode.

Movie S7. Path deflection of a Hg droplet (m = 200 mg) moving in an electric field using the galvanostatic condition (I = 3.0 mA) between the parallel electrodes. The domain was filled with a solution of sulfuric acid (c = 25 mM) and a piece of iron (III) chloride ($FeCl_3$) crystal was placed in the center of the container. The experiment started when the dissolved salt created a radially symmetric gradient of ions around the crystal. The depth of the solution in the domain was ~ 5 mm. The direction of the electric field was changed whenever the droplet arrived at the cathodes.

Movie S8. Refraction of the path of a Hg droplet (m = 80 mg) moving in an electric field through a boundary separating two different conductivity regions using the galvanostatic condition (I = 25 mA). The domain was filled with the solution of sulfuric acid (c = 25 mM). The depths of the solution was 2 mm in the upper part, and 3 mm in the bottom part.
